# Photo Quiz: Persistent papules in an immunocompromised child

**DOI:** 10.1128/jcm.01393-25

**Published:** 2026-04-16

**Authors:** Brannon G. Broadfoot, Saradhi Mallampati, Salem M. Jackson, Bobby L. Boyanton

**Affiliations:** 1 Department of Pathology, University of Arkansas for Medical Sciences 12215, Little Rock, Arkansas, USA; 2 Department of Pathology, Arkansas Children’s Hospital, Little Rock, Arkansas, USA; 3 College of Medicine, University of Arkansas for Medical Sciences 155638, Little Rock, Arkansas, USA; Mayo Clinic Minnesota, Rochester, Minnesota, USA

## PHOTO QUIZ 

**Fig 1:**
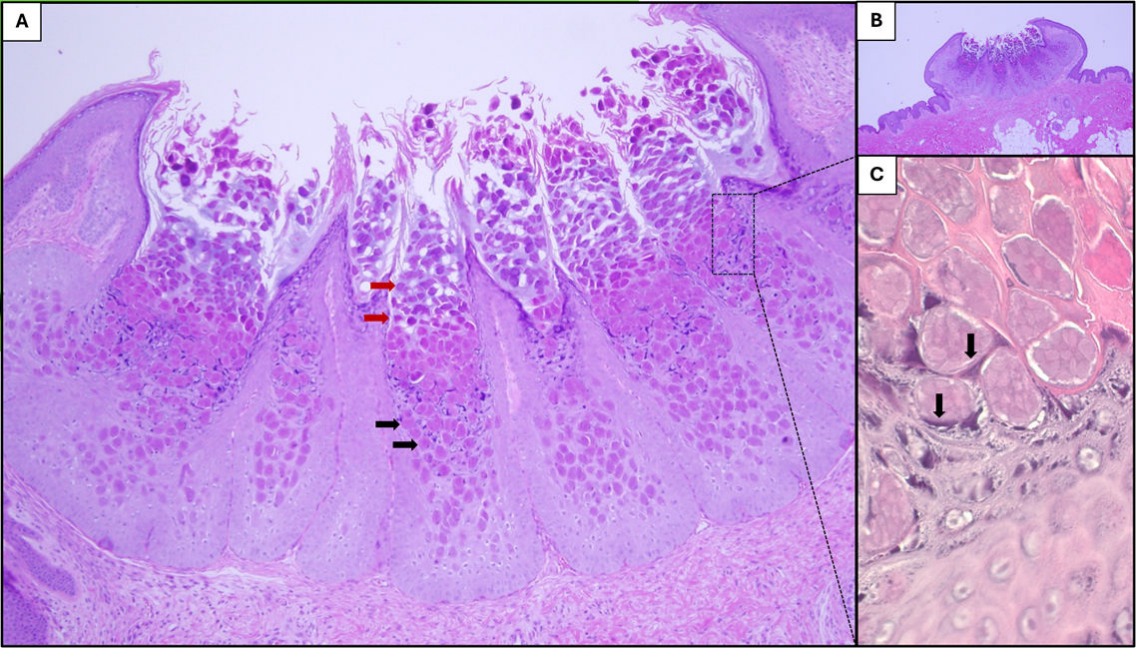
Skin biopsy, hematoxylin & eosin (magnification, 40× [**B**], 100× [**A**], 500× [**C**]). Dome-shaped papule exhibiting epidermal hyperplasia and central indentation/umbilication (panel **B**), containing numerous intracytoplasmic viral inclusions (Henderson-Patterson bodies) that transition from eosinophilic (panel **A**, black arrows) to basophilic (panel **A**, red arrows) during their ascent from the basal layer. Intracytoplasmic viral inclusions push aside the keratinocyte nuclei (panel **C**, black arrows).

An 8-year-old female presented to her Allergy and Immunology specialist for evaluation of a non-pruritic rash of 3 months’ duration. The rash was spreading despite treatment with an antifungal cream and mupirocin ointment prescribed by her primary care provider. Her medical history was significant for left lung hypoplasia and tetralogy of Fallot requiring surgical intervention as a neonate. At this time, single-nucleotide polymorphism chromosomal microarray analysis detected a 3.2 Mb deletion encompassing 43 contiguous genes consistent with chromosome 22q11.21 deletion syndrome (DiGeorge syndrome). As a result, she has chronic lymphopenia secondary to thymic hypoplasia. A concurrent complete blood count showed a white blood count of 9.46 × 10^9^/L (normal 5.00–14.50) with 76% neutrophils (normal 30%–55%), 15% lymphocytes (normal 30%–48%), 6% monocytes (normal 3%–8%), 2% eosinophils (normal 1%–4%), and 1% basophils (normal ≤1%); a hemoglobin of 128 g/L (normal 115–155 g/L); and a platelet count of 204 10^9^/L (normal 150–400). Her most recent lymphocyte subset analyses demonstrated an absolute CD3 count of 1.03 × 10^9^/L (normal 1.20–2.60 × 10^9^/L ), an absolute CD4 count of 0.58 × 10^9^/L (normal 0.65–1.50 × 10^9^/L ), and an absolute CD8 count of 0.30 × 10^9^/L (normal 0.37–1.1 × 10^9^/L ). The physical examination was significant for numerous tiny skin-colored papules on both legs; some appeared inflamed, and a few showed punctate white centers. Due to her immunocompromised state, a skin biopsy was obtained. The tissue was placed into a formalin fixative and processed for routine histopathologic examination (Fig. 1).

## ANSWER TO PHOTO QUIZ

The patient was infected with molluscum contagiosum virus (MCV), a human-specific double-stranded DNA poxvirus and the causative agent of molluscum contagiosum (MC). To date, four major MCV genotypes have been described. MCV-I and MCV-II are the most prevalent and associated with pediatric and adult infections, respectively (1). MCV-III and MCV-IV are less commonly encountered and currently appear geographically isolated to Asia and Australia (1). The virus is transmitted by skin-to-skin contact or via contaminated fomites. It manifests clinically as firm papules (usually 2–5 mm) with central umbilications that express a white waxy material (2, 3). In children, MC lesions are usually located on the face, torso, and extremities (excluding palms and soles); anogenital lesions are commonly observed in adults due to sexual transmission (2). The clinical appearance of MC lesions is diagnostic in most cases (2, 3). However, biopsy may be warranted to differentiate MC from cutaneous cryptococcosis or histoplasmosis, herpes simplex virus, human papillomavirus, monkeypox (mpox), varicella zoster virus, herpes simplex viruses, and other skin/adnexal tumors, especially in immunocompromised patients (3). Characteristic histological features include molluscum bodies, also known as Henderson-Patterson bodies, located in the enlarged superficial cells of the epidermis. These inclusion bodies, comprised of MCV particles, form within cytoplasmic vacuoles during viral replication (4, 5). In contrast, histologic findings of orthopoxvirus infections (e.g., cowpox, mpox, vaccinia, and variola) include ballooning degeneration of keratinocytes that contain eosinophilic intracytoplasmic inclusions (Guarnieri bodies) and occasionally ground glass-appearing keratinocyte nuclei admixed with a dense inflammatory cell infiltrate within the upper epidermal layers (6, 7). Although histopathological evaluation may aid in diagnosis, orthopoxvirus infections, including mpox, are typically confirmed using nucleic acid amplification assays such as real-time PCR (8).

DiGeorge or velocardiofacial syndrome (OMIM# 188400) is due to a heterozygous deletion in the chromosome 22q11.2 region (9). Clinical features observed in affected individuals include cardiac malformations, palatal abnormalities, immune deficiency, developmental delays, distinct facial features, psychiatric disorders, hypoparathyroidism, and other systemic anomalies. Symptoms range in severity from mild to severe, depending upon the size of the deletion and the respective genes involved (9). Haploinsufficiency of the *TBX1* gene within the deleted region contributes to an immunodeficient state via impaired T-cell production, leading to recurrent sinusitis, otitis media, or lower respiratory infections (9). As a consequence of T-cell lymphopenia, patients are at increased risk for atopy and autoimmune diseases, including juvenile rheumatoid arthritis and idiopathic thrombocytopenic purpura (9). This immunocompromised state predisposes affected individuals to a variety of infections, including extensive MC skin lesions, as observed in our patient (9).

MC is a self-limited disease that resolves within a few months in immunocompetent individuals but can be prolonged in those with compromised immune systems (3, 10). Treatment options include cryosurgery, curettage, podophyllin, cantharidin, tretinoin, cimetidine, potassium hydroxide, pulsed dye laser, imiquimod, and cidofovir. The side effects of each treatment modality, if clinically indicated, must be considered in the context of the patients’ medical condition (3). For individuals with lesions that (i) are extensive or located in atypical sites, (ii) persist greater than 6–9 months, and/or (iii) are treatment refractory, an underlying immunologic disorder should be considered (e.g., human immunodeficiency virus, iatrogenic immunosuppression, or primary immunodeficiency) (11). For our case, the patient and parents were counseled about the etiology of MC and how to prevent further individual spread and household transmission. One month later, interval improvement of the MC lesions was noted without pharmacologic intervention. The patient was referred to dermatology for continuity of care if the MC lesions had not resolved within 6 months.
